# Ancient Genetic Signatures of Orang Asli Revealed by Killer Immunoglobulin-Like Receptor Gene Polymorphisms

**DOI:** 10.1371/journal.pone.0141536

**Published:** 2015-11-13

**Authors:** Hanis Z. A. NurWaliyuddin, Mohd N. Norazmi, Hisham A. Edinur, Geoffrey K. Chambers, Sundararajulu Panneerchelvam, Zainuddin Zafarina

**Affiliations:** 1 Human Identification/DNA Unit, School of Health Sciences, Universiti Sains Malaysia, Health Campus, Kelantan, Malaysia; 2 Institute for Research in Molecular Medicine, Universiti Sains Malaysia, Health Campus, Kelantan, Malaysia; 3 School of Biological Sciences, Victoria University of Wellington, Wellington, New Zealand; 4 Malaysian Institute of Pharmaceuticals and Nutraceuticals, National Institutes of Biotechnology Malaysia, Ministry of Science, Technology and Innovation, Penang, Malaysia; Karolinska Institutet, SWEDEN

## Abstract

The aboriginal populations of Peninsular Malaysia, also known as Orang Asli (OA), comprise three major groups; Semang, Senoi and Proto-Malays. Here, we analyzed for the first time *KIR* gene polymorphisms for 167 OA individuals, including those from four smallest OA subgroups (Che Wong, Orang Kanaq, Lanoh and Kensiu) using polymerase chain reaction-sequence specific primer (PCR-SSP) analyses. The observed distribution of *KIR* profiles of OA is heterogenous; Haplotype *B* is the most frequent in the Semang subgroups (especially Batek) while Haplotype *A* is the most common type in the Senoi. The Semang subgroups were clustered together with the Africans, Indians, Papuans and Australian Aborigines in a principal component analysis (PCA) plot and shared many common genotypes (*AB6*, *BB71*, *BB73* and *BB159*) observed in these other populations. Given that these populations also display high frequencies of Haplotype *B*, it is interesting to speculate that Haplotype *B* may be generally more frequent in ancient populations. In contrast, the two Senoi subgroups, Che Wong and Semai are displaced toward Southeast Asian and African populations in the PCA scatter plot, respectively. Orang Kanaq, the smallest and the most endangered of all OA subgroups, has lost some degree of genetic variation, as shown by their relatively high frequency of the *AB2* genotype (0.73) and a total absence of *KIR2DL2* and *KIR2DS2* genes. Orang Kanaq tradition that strictly prohibits intermarriage with outsiders seems to have posed a serious threat to their survival. This present survey is a demonstration of the value of *KIR* polymorphisms in elucidating genetic relationships among human populations.

## Introduction

Human cells are equipped with markers of identity known as human leukocyte antigens (HLA) which are expressed on the cell surface and encoded by genes within the major histocompatibility complex (MHC) on chromosome 6. Virus infections and cancers may reduce the quantity of HLA relative to healthy cells [1]. Abnormal or reduced expression of HLA ligands can be detected by surface receptors on natural killer (NK) cells which then proceed to destroy the target cell. The NK lymphocytes are thus involved in the innate immune system and participate in adaptive immune responses [2, 3]. Various inhibitory and activating receptors are expressed on the surface of NK cells including killer immunoglobulin-like receptors (KIR), leukocyte-associated immunoglobulin-like receptor-1, CD16 and CD94. These receptors integrate intracellular signals that trigger NK cells to become cytotoxic or to produce cytokines and chemokines to lyse stressed and abnormal cells [4]. The *KIR* glycoproteins that impart specificity to NK cells are encoded by highly polymorphic and homologous genes on chromosome 19q13.4 [5] and have significant roles in human health [6] including transplantation compatibility [7] and pathogenesis of infectious [8] and autoimmune [9] diseases. The *KIR* genes with long cytoplasmic tail (L) carry immunoreceptor tyrosine-based inhibitory motifs and transmit inhibitory signals. In contrast, *KIR* genes with short cytoplasmic tail (S) express immunoreceptor tyrosine-based activating motifs to initiate NK cells activity by interaction with adaptor molecules [10, 11]. There are 13 *KIR* genes loci and two pseudogenes (*KIR2DP1* and *KIR3DP1)* that have been identified; six genes code for activating receptors (*KIR2DS1*, *KIR2DS2*, *KIR2DS3*, *KIR2DS4 KIR2DS5* and *KIR3DS1*), eight genes code for inhibitory receptors (*KIR2DL1*, *KIR2DL2*, *KIR2DL3*, *KIR2DL5A*, *KIR2DL5B*, *KIR3DL1*, *KIR3DL2* and *KIR3DL3*) and the product of one gene (*KIR2DL4*) transduces both types of signals. The *KIR2DL2* and *KIR2DL3* are encode by the same gene, as are for *KIR3DL1* and *KIR3DS1* [12, 13].

The *KIR* genotypes are determined by the combination of *KIR* genes present in an individual. They are organized into *A* and *B* haplotypes based on the number and type of *KIR* genes. Both haplotypes consist of four framework genes:- *KIR2DL4*, *KIR3DL2*, *KIR3DL3* and *KIR3DP1*. Haplotype *B* is recognized by the presence of one or more of the following genes; *KIR2DL2*, *KIR2DL5*, *KIR3DS1*, *KIR2DS1*, *KIR2DS2*, *KIR2DS3* and *KIR2DS5* [14]. Haplotype *A* is less variable and is classified by the presence of fixed inhibitory receptors (*KIR2DL1*, *KIR2DL3*, *KIR3DL1*) along with the activating (*KIR2DS4* and *KIR2DL4*) receptors [14].

Recently, there has been a substantial increase in the number of *KIR* genotypes and alleles reported in various population genetic studies. At present, 553 unique *KIR* genotypes have been recorded among 151 populations in Allele Frequency Net Database [15] and 753 *KIR* alleles are available in the Immuno Polymorphism Database [16]. Compilation of population studies reveal extensive *KIR* genotype variability across ethnically defined populations; North-East Asian regions such as China [17] and Japan [18] have high frequencies of Haplogroup *A* while African [19, 20], Indian [21, 22], Australian Aborigines [23] and Papuan [24] populations exhibit high frequencies of Haplogroup *B*. It has been suggested that human *KIR* genotype diversity is the accumulated consequence of numerous and successive selective episodes by different pathogens on human NK-cell responses [25]. Here, we are interested to study the *KIR* gene contents of the aboriginal people in Peninsular Malaysia locally known as Orang Asli (OA). This may provide some preliminary information on the different environmental challenges of the OA settlements compared with other locations. It is also interesting to determine whether *KIR* distribution in the OA are predominantly of Haplotype *A* or *B* in order to help delineate their genetic relationship and migration history in this region. In addition, this study may reveal new *KIR* genotype. No previous molecular population genetic studies involving the OA have included *KIR* analysis.

Currently, there are more than 178,000 OA individuals living in Peninsular Malaysia which represent ~0.5% of Malaysian population. The OA can be classified into three major groups; Semang, Senoi and Proto-Malays and each of the major group is further divided into six subgroups based on their language, physical appearance, culture and economic activities. The Semang were, until recently, hunter-gatherers, while the Senoi still practice slash and burn farming [26]. The Proto-Malays were previously seafarers, but are now mostly fishermen and merchants [27]. The groups that express the negrito phenotypes (i.e Batek, Jahai, Kensiu, Kintaq, Mendriq and Lanoh) are historically classified as Semang. They are associated with the first wave of modern humans travelling out of Africa (~25,000 to ~60,000 years ago) and undoubtedly are the descendants of the earliest settlers in Peninsular Malaysia [28, 29]. They are mutually related to the negrito peoples in the Philippines and Indonesia as well as the Australian Aborigines and indigenous tribes of Papua New Guinea and West Irian Jaya. Physically, they are of short-stature, have broad noses, low cheekbones, frizzy hair and dark skin colour. The Semang population only numbered around 5,000 individuals and currently live in small tribes within the low-land rainforests at the northern part of Peninsular Malaysia (Ministry of Rural and Regional Development; http://www.rurallink.gov.my). The Semang speak Austro-Asiatic (Aslian) language, a branch of Mon-Khmer language family, which is believed to be introduced via contact with the Senoi people.

The Senoi were involved in the second wave of prehistoric migration, which believed to have originated from the Indochina region. They migrated to Peninsular Malaysia during the Neolithic period ~7,000 years ago [29, 30]. The Senoi is the largest group of OA in Peninsular Malaysia (~98,000 individuals) and consist of six subgroups; Che Wong, Jahut, Mahmeri, Semaq Beri, Semai and Temiar. Their physical features differ from the Semang by having wavy hair, more pronounced cheekbones, and lighter skin colour. Their settlements are scattered across the high lands of central Peninsular Malaysia [30].

The Proto-Malays were proposed as the first wave of Mongoloid population expansion into the Islands of Southeast Asia [31]. Archaeological and linguistic data suggested that Proto-Malays are the descendants of Austronesian speaking voyagers who migrated from Taiwan through the Philippines to Borneo, Sulawesi, Java and finally settled in the coastal region of Peninsular Malaysia between ~6,000 and ~2,500 years ago [32, 33]. There are about 75,000 individuals who belong to the six subgroups of Proto-Malays; Orang Kanaq, Orang Kuala, Orang Seletar, Jakun, Semelai and Temuan, currently living in Peninsular Malaysia (Ministry of Rural and Regional Development; http://www.rurallink.gov.my). Their physical appearance, language (Malayo-Polynesian) and lifestyle are closely similar to modern Malays (Deutro-Malays). Most of the Proto-Malays are knowledgeable in oceanography and their settlements are concentrated along the coastal areas of southern Peninsular Malaysia.

At present, most of the OA subgroups remain semi-isolated. The Malaysian government has relocated many settlements to implement socio-economic development and enhance their societal prospects, while trying to preserve their traditional knowledge and heritage. Despite the intervention plans for the OA populations, which include several strategies to increase population numbers, Kensiu, Che Wong and Orang Kanaq continue to have only slow, if any, population growth. It is reported that Kensiu and Orang Kanaq showed population growth rate of only ~0.25% per year between 1998 to 2008 and zero population growth for the past 7 years.

To date, there are no *KIR* gene-content data available for any OA population and findings from the present study are expected to become increasingly important due to the increasing number of studies showing associations between specific *KIR* genotypes and various diseases such as diabetes mellitus [34], hepatitis B [35] and malaria [36], diseases that are also common in OA [37–39]. Our survey focuses on the subgroups with particularly small population number; Lanoh and Kensiu (Semang), Che Wong (Senoi) and Orang Kanaq (Proto-Malays), which have not shown any significant increase in numbers for the past 10 years (Ministry of Rural and Regional Development; http://www.rurallink.gov.my). We also include Batek (Semang) and Semai (Senoi), two of the largest OA subgroups in order to compare the *KIR* genes diversity between OA subgroups of various sizes in Peninsular Malaysia.

## Materials and Methods

### Study Populations

A total of 91 individuals from Semang (26 Lanoh, 27 Batek and 38 Kensiu), 65 individuals from Senoi (28 Che Wong and 37 Semai) and 11 individuals from Proto-Malays (Orang Kanaq) subgroups were recruited from six settlements in north (Lanoh, Batek and Kensiu at Lenggong, Gua Musang and Baling, respectively), central (Che Wong and Semai at Kuala Gandah and Kuala Lipis, respectively) and south (Orang Kanaq at Kota Tinggi, Johor) of Peninsular Malaysia. We analyzed the OA subgroups independently because they are not a homogenous group. They speak their own languages, practice individual cultures and regard themselves as different from one another. The participants were interviewed prior to recruitment into this study in order to establish the authenticity of their ancestral lineages. They were then divided into related and ‘unrelated’ groups based on the reported family pedigrees. Due to the small population size and limited knowledge of family pedigrees among some participants, we have to assume that all samples in each OA subgroup are related to some greater or lesser degree and further that the ‘unrelated’ group (15 Lanoh, 19 Batek, 22 Kensiu, 16 Che Wong, 29 Semai and 7 Orang Kanaq), is effectively a sub-set of the related group which excludes siblings. Number of participants in this study is also limited by the fact that many OA have admixed intermarriage backgrounds within the last two generations. Nonetheless, we have managed to identify those few individuals in each subgroup without mixed lineages for at least three generations. The selected participants were fully informed about this research project prior to sample collection. Their consent was obtained either by signing or thumbprint on the participant's information and consent form. Blood samples were collected by registered medical officers. Ethical approvals were obtained from Universiti Teknologi MARA and Universiti Sains Malaysia Human Ethics Committee (FWA Reg. No: 00007718; IRB Reg. No: 00004494).

### DNA Extraction and KIR Genotyping

Genomic DNA was extracted using the Qiagen Dneasy® Blood & Tissue Kit following the manufacturer’s protocol (www.qiagen.com). The *KIR* genotyping was carried out using 16 independent PCR-SSP reaction mixtures as described by Vilches et al. [40] for all 14 *KIR* genes and two pseudogenes. This method does not distinguished between *KIR2DL5A* and *KIR2DL5B* and we chose to report *KIR2DL2*/*KIR2DL3* and *KIR3DL1*/*KIR3DS1* genes separately since they show different frequencies in the tested populations. Amplification products were electrophoresed on 2.0% agarose gels for 40 minutes and visualized using a digital gel documentation system (Vilber Lourmat, Deutschland GmbH, Eberhardzell, Germany).

### Statistical Analysis

The observed *KIR* gene frequencies (*F*) were calculated using the direct counting method. The estimated gene frequencies (*gF*), assuming Hardy-Weinberg (HW) proportions, were computed using Bernstein’s formula: $gF=\;1\;-\sqrt{\left(1-F\right)}$, which were used in later analyses and for populations comparisons. The prediction of *KIR* genotypes following Rajalingam et al. [21] and *KIR2DL2*, *KIR2DL3*, *KIR3DL1* and *KIR3DS1* were considered as a single *KIR* gene locus. The *AA* genotypes carry only *KIR3DL3*, *KIR2DL3*, *KIR2DL1*, *KIR2DP1*, *KIR3DP1*, *KIR2DL4*, *KIR3DL1*, *KIR2DS4* and *KIR3DL2* genes whereas *BB* genotypes were recognized by the absence of any of the following four genes; *KIR2DL1*, *KIR2DL3*, *KIR3DL1* and *KIR2DS4*. All other genotypes were considered as heterozygous *AB*. The *KIR* genotype number (ID) was assigned by Allele Frequency Net Database [15]. Haplotype *A* and *B* frequencies were counted using the standard formula: haplotype *A* = (2nAA + nAB)/2N and haplotype *B* = (2nBB + nAB)/2N, where nAA, nAB and nBB are the numbers of individuals with genotype profile *AA*, *AB* and *BB*, respectively, and N is the sample size of each subgroup. Scatter plot of haplotype *B* vs *A* frequency was constructed to assess the genetic relationship of the OA subgroups with other global populations. Next, HW analysis was applied to test if variation in the OA subgroups are at genetic equilibrium using the conventional chi-square (χ^2^) goodness of fit test [41]. Subgroups with χ^2^ value < 3.841 are considered to be in HW equilibrium. Principal component analysis (PCA) was used to demonstrate genetic affinity among the populations compared. The PCA was constructed using Multivariate Statistical Package software v3.22 (Kovach Computing Services, UK; http://www.kovcomp.com/mvsp) based on a set of seven *gF* values (i.e. those for *KIR3DL1*, *KIR2DL1*, *KIR2DL3*, *KIR2DL2*, *KIR3DS1*, *KIR2DS1* and *KIR2DS2*) which are available for all of the selected reference populations. Linkage disequilibrium (LD) was performed using XLSTAT software to measure the possibility that any two particular *KIR* genes were inherited together in each OA subgroup. A value toward ‘1.00’ shows high tendency of two genes to be inherited together with *p*-value <0.05 as a level of significance. Homogeneity tests were conducted to analyze the distribution of single gene between two populations using χ^2^ with Yates' correction by using Graphpad software (http://www.graphpad.com) with *p*-values <0.05 as level of significance.

## Results

**Fig 1:**
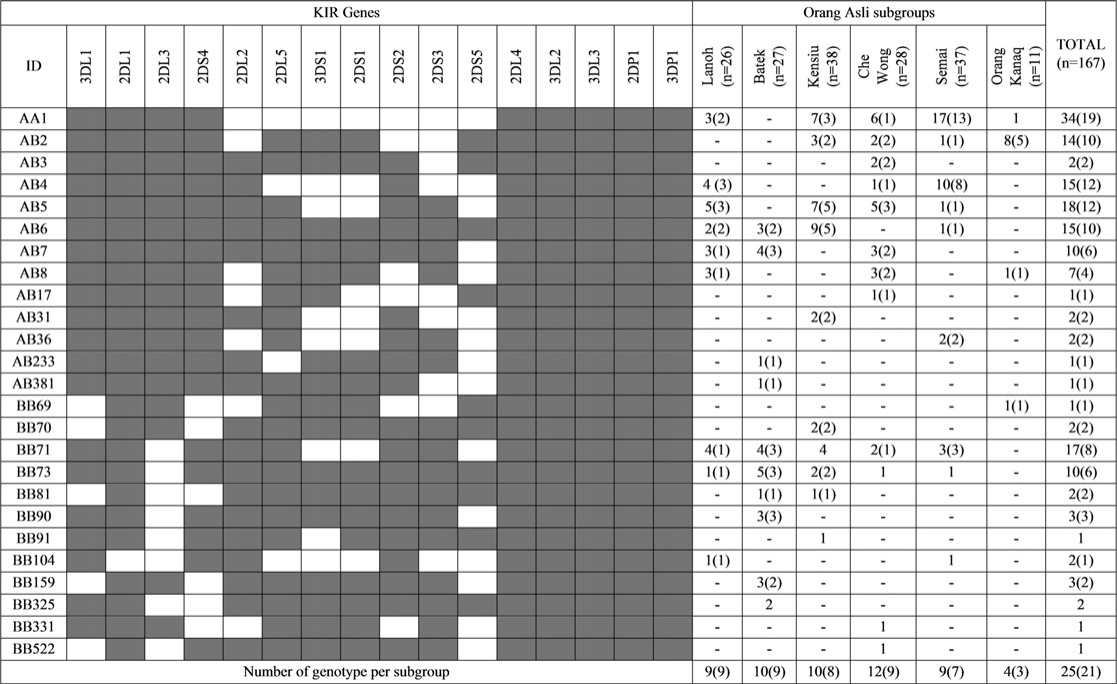
The *KIR* genotype profiles of six OA subgroups in Peninsular Malaysia. ID is the genotype number assigned by Allele Frequency Net Database [15]. Numbers in brackets represent the ‘unrelated sample’ of OA subgroups.

All of the 16 *KIR* genes presently known were detected in all of the OA subgroups, except for *KIR2DL2* and *KIR2DS2* genes which are completely absent in Orang Kanaq (Table 1). There were also relatively low frequencies of *KIR2DS3* (0.05) in Orang Kanaq and *KIR2DS1*, *KIR2DS5* and *KIR3DS1* genes (0.04 each) in Semai. There were high frequencies of *KIR* inhibitory genes (0.32–1.00) in Lanoh, Kensiu and Che Wong. On the contrary, Batek and Orang Kanaq have a balance distribution of both functional types of *KIR* genes. We have also examined the frequencies of *KIR* genes in the ‘unrelated’ OA subgroups, but only minor differences were observed when compared with the corresponding total OA subgroups (Table 1 and S1 Table). The framework genes; *KIR2DL4*, *KIR3DL2*, *KIR3DL3* and *KIR3DP1* together with one pseudogene *KIR2DP1* were present in all individuals. Fifteen individuals were observed to have all 16 *KIR* genes; 9 Kensiu, 3 Batek, 2 Lanoh and 1 individual from Semai (Fig 1).

**Table 1:** Observed (*F*) and estimated (*gF*) *KIR* genes frequencies for OA subgroups.

Population	Semang						Senoi				Proto-Malays	
Subgroup	Lanoh (n = 26)		Batek (n = 27)		Kensiu (n = 38)		Che Wong (n = 28)		Semai (n = 37)		Orang Kanaq (n = 11)	
KIR gene	*F*	*gF*	*F*	*gF*	*F*	*gF*	*F*	*gF*	*F*	*gF*	*F*	*gF*
3DL1	1.00	1.00	0.85	0.61	0.91	0.70	0.96	0.80	1.00	1.00	0.91	0.70
2DL1	0.96	0.80	1.00	1.00	1.00	1.00	1.00	1.00	0.97	0.83	1.00	1.00
2DL3	0.77	0.52	0.44	0.25	0.79	0.54	0.86	0.63	0.86	0.63	1.00	1.00
2DS4	1.00	1.00	0.78	0.53	0.91	0.70	0.96	0.80	1.00	1.00	0.91	0.70
2DL2	0.77	0.52	1.00	1.00	0.68	0.43	0.54	0.32	0.46	0.27	0.00	0.00
2DL5	0.69	0.44	0.96	0.80	0.76	0.51	0.75	0.50	0.24	0.13	0.91	0.70
3DS1	0.35	0.19	0.85	0.61	0.47	0.27	0.50	0.29	0.08	0.04	0.91	0.70
2DS1	0.35	0.19	0.85	0.61	0.47	0.27	0.46	0.27	0.08	0.04	0.91	0.70
2DS2	0.77	0.52	1.00	1.00	0.68	0.43	0.54	0.32	0.51	0.30	0.00	0.00
2DS3	0.69	0.44	0.96	0.80	0.65	0.41	0.57	0.34	0.22	0.12	0.09	0.05
2DS5	0.12	0.06	0.41	0.23	0.47	0.27	0.21	0.11	0.08	0.04	0.82	0.58
2DL4	1.00	1.00	1.00	1.00	1.00	1.00	1.00	1.00	1.00	1.00	1.00	1.00
3DL2	1.00	1.00	1.00	1.00	1.00	1.00	1.00	1.00	1.00	1.00	1.00	1.00
3DL3	1.00	1.00	1.00	1.00	1.00	1.00	1.00	1.00	1.00	1.00	1.00	1.00
2DP1	1.00	1.00	1.00	1.00	1.00	1.00	1.00	1.00	1.00	1.00	1.00	1.00
3DP1	1.00	1.00	1.00	1.00	1.00	1.00	1.00	1.00	1.00	1.00	1.00	1.00

*F* is using direct counting method based on the presence of *KIR* gene in the subgroup.

*gF* is derived from *F* by using formula $gF=\;1-\sqrt{\left(1-F\right)}$.

A total of 25 *KIR* genotype profiles were discovered among the 167 OA individuals, (Fig 1 and Table 2). The distribution of unique and shared genotypes is presented in Table 2. The *BB71* and *BB73* genotypes were present in all OA subgroups except for Orang Kanaq. The Che Wong subgroup has the highest number of *KIR* genotypes (12 *KIR* profiles among 28 individuals), followed by Batek (10 *KIR* profiles among 27 individuals), and the lowest is Semai (9 *KIR* profiles among 37 individuals). The *AB2* genotype is the most common genotype in Orang Kanaq (0.73) while the genotype *AA1* (0.46) and *AB4* (0.27) are the most frequent genotypes in Semai (Fig 1 and Table 2).

**Table 2:** The distribution of unique and shared *KIR* genotypes between OA subgroups.

	Singleton					Doubles	Triples	Quadruplets	Pentuples
	Batek	Kensiu	Che Wong	Semai	Orang Kanaq				
	(AB233;1)	(AB31;2)	(AB3;2)	(AB36;2)	(BB69;1)	BK/KS (BB81;2)	LH/CW/SM (AB4;15)	KS/CW/SM/OK (AB2;14)	LH/KS/CW/SM/OK
	(AB381;1)	(BB70;2)	(AB17;1)			LH/SM (BB104;2)	LH/BK/CW (AB7;10)	LH/KS/CW/SM (AB5;18)	(AA1;34)
	(BB90;3)	(BB91;1)	(BB331;1)				LH/CW/OK (AB8;7)	LH/BK/KS/SM (AB6;15)	LH/BK/KS/CW/SM
	(BB159;3)		(BB522;1)						(BB71;17, BB73;10)
	(BB325;2)								
Total	(5;10)	(3;5)	(4;5)	(1;2)	(1;1)	(2;4)	(3;32)	(3;47)	(3;61)

Singleton indicates the unique genotype that present in only one studied OA subgroup. Doubles, Triples, Quadruplets and Pentuples show the genotype is shared by two, three, four and five OA subgroups. Genotype numbers are assigned by Allele Frequency Net Database [15]. BK = Batek, CW = Che Wong, SM = Semai, LH = Lanoh, KS = Kensiu, OK = Orang Kanaq.

**Fig 2:**
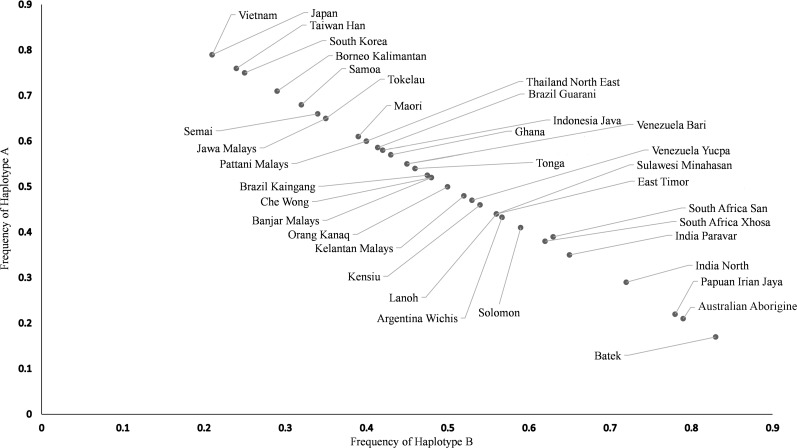
Distribution of *KIR* haplotype *A* and *B* frequencies among global populations. All *KIR* haplotypes frequencies used are listed in S5 Table.

The *KIR* genotype and haplotype frequencies are presented in Table 3. Semai showed the highest frequency of Haplotype *A* (0.66), followed by Che Wong (0.52). Haplotype *B* is predominant in all Semang subgroups; Batek (0.83), Lanoh (0.56) and Kensiu (0.54). The HW analysis indicated that all OA subgroups are in equilibrium except for Orang Kanaq (S2A and S2B Table). The distribution of haplotype *A* and *B* frequencies among OA subgroups and worldwide populations is displayed in Fig 2. Batek has the highest frequency of haplotype *B* when compared with other world populations reported so far. On the other hand, the Semai are closer to Northeast Asian populations which consistently show high frequencies of haplotype *A*.

**Table 3:** *KIR* genotypes and haplotypes frequencies for OA subgroups.

Orang Asli subgroup	Genotype			Haplotype	
	AA	AB	BB	A	B
Lanoh (n = 26)	0.12	0.65	0.23	0.44	0.56
Batek (n = 27)	0.00	0.33	0.67	0.17	0.83
Kensiu (n = 38)	0.18	0.55	0.26	0.46	0.54
Che Wong (n = 28)	0.21	0.61	0.18	0.52	0.48
Semai (n = 37)	0.46	0.41	0.14	0.66	0.34
Orang Kanaq (n = 11)	0.09	0.82	0.09	0.50	0.50

The prediction of *AA*, *AB* and *BB* genotypes following Rajalingam et al. [21]. Haplotypes frequencies are calculated using the standard formula (see Materials and Methods).

The LD analyses between pairs of *KIR* genes in OA subgroups are listed in S3A–S3L Table. Any *KIR* genes that are completely fixed or absent in the sample group were excluded from the LD analyses. In general, there were strong positive association between the *KIR2DL2-KIR2DS2* and *KIR2DS1*-*KIR3DS1* pairs in all six OA subgroups. Strong positive associations were also detected between pairs of *KIR2DL5*-*KIR2DS3* in Lanoh, *KIR2DS4-KIR3DL1* in Batek, Kensiu and Orang Kanaq and *KIR2DS5-KIR2DS1*/*KIR3DS1* in Semai and Orang Kanaq.

The homogeneity tests between pairs of OA subgroups are shown in S4A–S4F Table. All six OA subgroups showed no significant differences for *KIR2DL1* and *KIR3DL1* genes. However, several genes in Batek (i.e. *KIR2DL2*, *KIR2DL3*, *KIR2DS1*, *KIR2DS2*, *KIR2DS3* and *KIR3DS1*), Semai (i.e. *KIR2DL5*, *KIR2DS1*, *KIR2DS3* and *KIR3DS1*) and Orang Kanaq (i.e. *KIR2DL2*, *KIR2DS2*, *KIR2DS3* and *KIR2DS5*) were significantly different compared with other OA subgroups.

**Fig 3:**
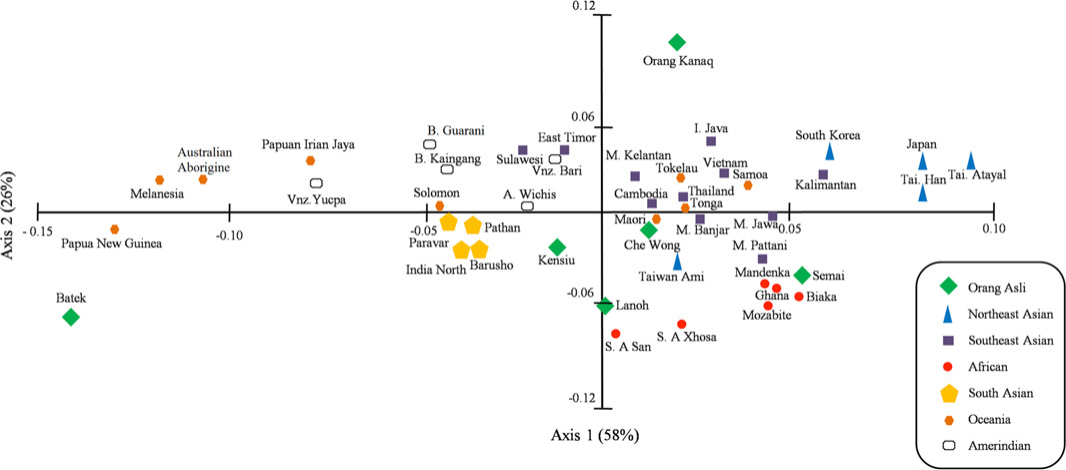
Principal component analysis plot shows the distribution of global populations based on *KIR* genes. All estimated *KIR* genes frequencies (*gF*) of the populations used are listed in S5 Table. A = Argentina; B = Brazil; I = Indonesia; M = Malay; S. A = South Africa; Tai = Taiwan; Vnz = Venezuela.

In the PCA plot, all reference populations are distributed according to their ethnogeographical regions; Northeast Asian, Southeast Asian, South Asian, African, Amerindian and Oceania (Fig 3). The Semang subgroups showed some degree of genetic affinity to the Africans, South Asians, Papuans and Australia Aborigines as Lanoh, Kensiu and Batek are plotted in between these populations. Che Wong and Semai, the subgroups of Senoi are plotted closer to the Southeast Asian and African populations, respectively. In contrast, Orang Kanaq is well separated from other reference datasets including the Austronesian speaking populations.

## Discussion

Malaysia is a multiethnic country that received multiple sucessive waves of more or less ancient migrations giving rise to Semang, Senoi and Proto-Malay subgroups. This followed by re-settlements of various Austronesian speakers (Malayo-Polynesian) from neigbouring islands including Jawa (Java), Banjar (Kalimantan), Bugis (Sulawesi) and Minangkabau (Sumatera) Malays [42, 43] and an influx of traders and labourers during Malacca Sultanate (1402–1511). British colonization (1824–1956) has added further genetic diversity to the population in more recent times. Thus, any genetic study of people in Malaysia is complicated as it involves these repeated waves of migration and/or admixture of people from different genetic backgrounds.

Our findings showed that the Semang subgroups (Lanoh, Kensiu and Batek) exhibit high frequencies of *KIR* haplotype *B* (see Fig 1 and Table 3) and share three *KIR* genotypes; *AB6* (14 individuals), *BB71* (12 individuals) and *BB73* (8 individuals) that were previously reported as the most common genotypes in African and Indian populations [19–22] (Table 2). They were also plotted closest to African, Indian, Papuan and Australian Aborigine populations in the haplotype fraction and PCA graphs (Figs 2 and 3). This is an evidence of their ancient genetic affinities as part of the first wave out of Africa movement by modern human travelling along southern coast of Arabian Peninsula towards India, Southeast Asia (Peninsular Malaysia) and finally arriving in Australia and Papua New Guinea [44, 45]. Other genetic signatures of the ancient lineage in Semang is demonstrated by the presence of *KIR* genotype *BB159* in Batek, which is also a common *KIR* genotype in Africans, Indians, Australian Aborigines and Papuans [15,22].

Senoi is the largest OA population in Peninsular Malaysia and frequently migrate in small groups from one settlement to another [46]. This practice may reduce genetic variation in the Senoi, especially for the Semai subgroup where *AA1* and *AB4* represent more than 70% of all their observed *KIR* genotype profiles (see Fig 1). Overall, the two Senoi subgroups (Semai and Che Wong) were observed to have high frequencies of haplotype *A* (Table 3). Che Wong is plotted closer to the Southeast Asia populations (see Fig 3) and is entirely consistent with their proposed origins from Southern China [29] whereas the Semai is displaced towards African populations. The genetic differences between Senoi subgroups (Che Wong and Semai) are probably due to different admixture level with the earlier Semang groups that carry the negrito phenotype. This observation indicates the gene flow from Semang have significantly changed the genetic composition of Semai. Their ancestral genepool are retained by being isolated in the deep rainforest of Banjaran Titiwangsa compared with the smaller number of Che Wong individuals who are now frequently exposed to outsiders as their village, Kuala Gandah has been commercialized for tourism.

The Proto-Malays are suggested to be the introducer of Austronesian language into the Island of Southeast Asia. However, Orang Kanaq is significantly different from other Austronesian speaking populations and showing evidence of becoming a distinct population (Fig 3). They were also distant from the neighbouring Deutro-Malay subgroups [47], even though both speak similar language (Malayo-Polynesian) and express similar phenotypes. Their low population number and living in isolation might indirectly increase inbreeding and reduce their genetic diversity, as shown by the elevated *AB2* genotype frequency (~0.73), entire absence of *KIR2DL2* and *KIR2DS2* genes and relatively low frequency of *KIR2DS3* (Fig 1, Tables 1 and 2). All these factors might underlie the significant differences observed between Proto-Malays (Orang Kanaq) and Deutro-Malay subgroups in the present account of Austronesian origins and migration patterns (Fig 3 and [48]). In contrast, the Deutro-Malay subgroups form a major component of multiracial population of Malaysia and their genepools are becoming more diverse due to intermarriages with other ethnicities. However, this observation should be interpreted with caution due the small sample size in this study with only 11 individuals of Orang Kanaq analyzed. Unfortunately, we were unable to improve on this situation because there is only small number of Orang Kanaq still in existence (approximately 80 individuals only). The strict sampling selection criteria that we used has also contributed to the low number of samples obtained.

## Conclusions

Overall, comprehensive *KIR* gene-content datasets generated from the present survey provides essential knowledge on the genetic relationships within the OA subgroups and with other world populations. The observed distribution of *KIR* profiles of OA is heterogenous; Haplotype *B* is the most frequent in the Semang subgroups (especially Batek) while Haplotype *A* is the most common type in the Senoi. In the PCA plot, the Batek is grouped together with Africans, Indians, Papuans and Australia Aborigines, showing closer affinity to these populations. Given that these populations also display high frequencies of Haplotype *B*, it is interesting to speculate that relatively high frequencies of Haplotype *B* may be a general feature of ancient populations, which fits well with molecular age estimation made using mtDNA sequence data analysis [45]. In contrast, the Senoi who show high frequencies of Haplotype A could be linked to the Southern China populations. The Orang Kanaq (Proto Malays) do not seems to have any particular common haplotype which is similar to other Austronesian speakers in the region.

## Supporting Information

S1 Table Observed (*F*) and estimated (*gF*) *KIR* genes frequencies for ‘unrelated sample’ of OA subgroups. (DOC)

S2 Table HW analysis for ‘total sample’ (Table A) and ‘unrelated sample’ (Table B) of OA subgroups. e = expected sample; f = frequency; HW = Hardy-Weinberg; n = sample size; o = observed sample; *p* = p-value (*p*< 0.05 is considered significant); χ^2^ = chi-squared value (χ^2^ > 3.841 shows the subgroup is deviate from HW equilibrium).
(DOC)

S3 Table Linkage disequilibrium analyses between pairs of KIR genes in (a) total Lanoh, (b) ‘unrelated’ Lanoh, (c) total Batek, (d) ‘unrelated’ Batek, (e) total Kensiu, (f) ‘unrelated’ Kensiu, (g) total Che Wong, (h) ‘unrelated’ Che Wong, (i) total Semai, (j) ‘unrelated’ Semai, (k) total Orang Kanaq and (l) ‘unrelated’ Orang Kanaq. Correlation matrix values range between 1.00 to -1.00. The value toward ‘1.00’ shows high tendency of two genes inherited together with *p*-value <0.05 as level of significance.
(DOC)

S4 Table Homogeneity tests (*p*-values) between pairs of OA subgroups using (a) *KIR3DL1* and *KIR2DL1*, (b) *KIR2DL3* and *KIR2DS4*, (c) *KIR2DL2* and *KIR2DL5*, (d) *KIR3DS1* and *KIR2DS1*, (e) *KIR2DS2* and *KIR2DS3* and (f) *KIR2DS5* with *p*-values <0.05 as level of significance. C.C.,cannot be calculated. (DOC)

S5 Table *KIR* estimated genes (*gF*) and haplotypes frequencies for global populations. The datasets were used to construct Figs 2 and 3. C.C = cannot be calculated; F = frequency, N.T = not tested. (DOC)
