# Supplementary material for: Ancient Genetic Signatures of Orang Asli Revealed by Killer Immunoglobulin-Like Receptor Gene Polymorphisms
Source: PLoS One. 2015 Nov 13;10(11):e0141536. doi: 10.1371/journal.pone.0141536 (PMC4643969; doi:10.1371/journal.pone.0141536)
Supplement: S1 Table — (DOC) [file pone.0141536.s001.doc]

**S1 Table. Observed (*F*) and estimated (*gF*) *KIR* genes frequencies for ‘unrelated sample’ of OA subgroups.**

| Population | Semang | | | | | | Senoi | | | | Proto-Malays | |
| --- | --- | --- | --- | --- | --- | --- | --- | --- | --- | --- | --- | --- |
| Subgroup | Lanoh (n=15) | | Batek (n=19) | | Kensiu (n=22) | | Che Wong (n=16) | | Semai (n=29) | | Orang Kanaq (n=7) | |
|  |  | |  | |  | |  | |  | |  | |
| KIR gene | *F* | *gF* | *F* | *gF* | *F* | *gF* | *F* | *gF* | *F* | *gF* | *F* | *gF* |
| 3DL1 | 1.00 | 1.00 | 0.84 | 0.60 | 0.86 | 0.63 | 0.94 | 0.76 | 1.00 | 1.00 | 0.86 | 0.63 |
| 2DL1 | 0.93 | 0.74 | 1.00 | 1.00 | 1.00 | 1.00 | 1.00 | 1.00 | 1.00 | 1.00 | 1.00 | 1.00 |
| 2DL3 | 0.80 | 0.55 | 0.47 | 0.27 | 0.86 | 0.63 | 0.88 | 0.65 | 0.90 | 0.68 | 1.00 | 1.00 |
| 2DS4 | 1.00 | 1.00 | 0.84 | 0.60 | 0.86 | 0.63 | 1.00 | 1.00 | 1.00 | 1.00 | 0.86 | 0.63 |
| 2DL2 | 0.80 | 0.55 | 1.00 | 1.00 | 0.73 | 0.48 | 0.63 | 0.39 | 0.45 | 0.26 | 0.00 | 0.00 |
| 2DL5 | 0.60 | 0.37 | 0.95 | 0.78 | 0.82 | 0.58 | 0.88 | 0.65 | 0.28 | 0.15 | 1.00 | 1.00 |
| 3DS1 | 0.33 | 0.18 | 0.84 | 0.60 | 0.59 | 0.36 | 0.63 | 0.39 | 0.07 | 0.04 | 1.00 | 1.00 |
| 2DS1 | 0.33 | 0.18 | 0.84 | 0.60 | 0.55 | 0.33 | 0.56 | 0.34 | 0.07 | 0.04 | 1.00 | 1.00 |
| 2DS2 | 0.80 | 0.55 | 1.00 | 1.00 | 0.73 | 0.48 | 0.63 | 0.39 | 0.52 | 0.31 | 0.00 | 0.00 |
| 2DS3 | 0.60 | 0.37 | 0.95 | 0.78 | 0.68 | 0.43 | 0.56 | 0.34 | 0.24 | 0.13 | 0.14 | 0.07 |
| 2DS5 | 0.20 | 0.11 | 0.32 | 0.18 | 0.55 | 0.33 | 0.31 | 0.17 | 0.07 | 0.04 | 0.86 | 0.63 |
| 2DL4 | 1.00 | 1.00 | 1.00 | 1.00 | 1.00 | 1.00 | 1.00 | 1.00 | 1.00 | 1.00 | 1.00 | 1.00 |
| 3DL2 | 1.00 | 1.00 | 1.00 | 1.00 | 1.00 | 1.00 | 1.00 | 1.00 | 1.00 | 1.00 | 1.00 | 1.00 |
| 3DL3 | 1.00 | 1.00 | 1.00 | 1.00 | 1.00 | 1.00 | 1.00 | 1.00 | 1.00 | 1.00 | 1.00 | 1.00 |
| 2DP1 | 1.00 | 1.00 | 1.00 | 1.00 | 1.00 | 1.00 | 1.00 | 1.00 | 1.00 | 1.00 | 1.00 | 1.00 |
| 3DP1 | 1.00 | 1.00 | 1.00 | 1.00 | 1.00 | 1.00 | 1.00 | 1.00 | 1.00 | 1.00 | 1.00 | 1.00 |
