# Supplementary material for: Ancient Genetic Signatures of Orang Asli Revealed by Killer Immunoglobulin-Like Receptor Gene Polymorphisms
Source: PLoS One. 2015 Nov 13;10(11):e0141536. doi: 10.1371/journal.pone.0141536 (PMC4643969; doi:10.1371/journal.pone.0141536)
Supplement: S2 Table — e = expected sample; f = frequency; HW = Hardy-Weinberg; n = sample size; o = observed sample; p = p-value (p< 0.05 is considered significant); χ2 = chi-squared value (χ2 > 3.841 shows the subgroup is deviate from HW equilibrium). (DOC) [file pone.0141536.s002.doc]

**S2 Table. HW analysis for ‘total sample’ (Table A) and ‘unrelated sample’ (Table B) of OA subgroups.**

**Table A**

|  | Lanoh (n=26) | | | Batek (n=27) | | | Kensiu (n=38) | | |
| --- | --- | --- | --- | --- | --- | --- | --- | --- | --- |
| Genotype | o (f) | e (f) | HW analysis | o (f) | e (f) | HW analysis | o (f) | e (f) | HW analysis |
| AA | 0.12 | 0.20 |  | 0.00 | 0.03 |  | 0.18 | 0.21 |  |
| AB | 0.65 | 0.49 | χ²= 2.75 | 0.33 | 0.28 | χ²= 1.08 | 0.55 | 0.50 | χ²= 0.48 |
| BB | 0.23 | 0.31 | *p*= 0.10 | 0.67 | 0.69 | *p*= 0.30 | 0.26 | 0.29 | *p*= 0.49 |
|  |  |  |  |  |  |  |  |  |  |
|  | Che Wong (n=28) | | | Semai (n=37) | | | Orang Kanaq (n=11) | | |
| Genotype | o (f) | e (f) | HW analysis | o (f) | e (f) | HW analysis | o (f) | e (f) | HW analysis |
| AA | 0.21 | 0.27 |  | 0.46 | 0.44 |  | 0.09 | 0.25 |  |
| AB | 0.61 | 0.50 | χ²= 1.30 | 0.41 | 0.45 | χ²= 0.33 | 0.82 | 0.50 | χ²= 4.46 |
| BB | 0.18 | 0.23 | *p*= 0.25 | 0.14 | 0.11 | *p*= 0.57 | 0.09 | 0.25 | *p*= 0.03 |

Table B

|  | Lanoh (n=15) | | | Batek (n=19) | | | Kensiu (n=22) | | |
| --- | --- | --- | --- | --- | --- | --- | --- | --- | --- |
| Genotype | o (f) | e (f) | HW analysis | o (f) | e (f) | HW analysis | o (f) | e (f) | HW analysis |
| AA | 0.13 | 0.22 |  | 0.00 | 0.03 |  | 0.14 | 0.21 |  |
| AB | 0.67 | 0.50 | χ²= 1.73 | 0.37 | 0.30 | χ²= 0.97 | 0.64 | 0.50 | χ²= 1.77 |
| BB | 0.20 | 0.28 | *p*= 0.19 | 0.63 | 0.66 | *p*= 0.32 | 0.23 | 0.30 | *p*= 0.18 |
|  |  |  |  |  |  |  |  |  |  |
|  | Che Wong (n=16) | | | Semai (n=29) | | | Orang Kanaq (n=7) | | |
| Genotype | o (f) | e (f) | HW analysis | o (f) | e (f) | HW analysis | o (f) | e (f) | HW analysis |
| AA | 0.06 | 0.22 |  | 0.45 | 0.45 |  | 0.00) | 0.18 |  |
| AB | 0.81 | 0.50 | χ²= 6.38 | 0.45 | 0.44 | χ²= 0.01 | 0.86 | 0.49 | χ²= 3.94 |
| BB | 0.13 | 0.28 | *p*= 0.01 | 0.10 | 0.11 | *p*= 0.92 | 0.14 | 0.33 | *p*= 0.05 |

e = expexted sample; f = frequency; HW = Hardy-Weinberg; n = sample size; o = observed sample; *p* = p-value (*p*< 0.05 is considered significant); χ² = chi-squared value (χ² > 3.841 shows the subgroup is deviate from HW equilibrium).
