# Supplementary material for: Ancient Genetic Signatures of Orang Asli Revealed by Killer Immunoglobulin-Like Receptor Gene Polymorphisms
Source: PLoS One. 2015 Nov 13;10(11):e0141536. doi: 10.1371/journal.pone.0141536 (PMC4643969; doi:10.1371/journal.pone.0141536)
Supplement: S3 Table — Correlation matrix values range between 1.00 to -1.00. The value toward ‘1.00’ shows high tendency of two genes inherited together with p-value <0.05 as level of significance. (DOC) [file pone.0141536.s003.doc]

**S3 Table. Linkage disequilibrium analyses between pairs of KIR genes in (a) total Lanoh, (b) ‘unrelated’ Lanoh, (c) total Batek, (d) ‘unrelated’ Batek, (e) total Kensiu, (f) ‘unrelated’ Kensiu, (g) total Che Wong, (h) ‘unrelated’ Che Wong, (i) total Semai, (j) ‘unrelated’ Semai, (k) total Orang Kanaq and (l) ‘unrelated’ Orang Kanaq.** Correlation matrix values range between 1.00 to -1.00. The value toward ‘1.00’ shows high tendency of two genes inherited together with *p*-value <0.05 as level of significance.

**a. Linkage disequilibrium analyses between pairs of *KIR* genes in ‘total sample’ of Lanoh**

| KIR Genes | 2DL1 | 2DL3 | 2DL2 | 2DL5 | 3DS1 | 2DS1 | 2DS2 | 2DS3 | 2DS5 |
| --- | --- | --- | --- | --- | --- | --- | --- | --- | --- |
|  | Correlation Matrix (Pearson) | | | | | | | | |
| 2DL1 | - | 0.37 | -0.11 | 0.30 | 0.15 | 0.15 | -0.11 | 0.30 | 0.07 |
| 2DL3 | 0.07 | - | -0.30 | -0.17 | 0.21 | 0.21 | -0.30 | -0.17 | -0.09 |
| 2DL2 | 0.59 | 0.14 | - | 0.23 | -0.18 | -0.18 | 1.00 | 0.23 | 0.20 |
| 2DL5 | 0.14 | 0.41 | 0.26 | - | 0.49 | 0.49 | 0.23 | 1.00 | 0.24 |
| 3DS1 | 0.48 | 0.31 | 0.39 | 0.01 | - | 1.00 | -0.18 | 0.49 | 0.50 |
| 2DS1 | 0.48 | 0.31 | 0.39 | 0.01 | < 0.01 | - | -0.18 | 0.49 | 0.50 |
| 2DS2 | 0.59 | 0.14 | < 0.01 | 0.26 | 0.39 | 0.39 | - | 0.23 | 0.20 |
| 2DS3 | 0.14 | 0.41 | 0.26 | < 0.01 | 0.01 | 0.01 | 0.26 | - | 0.24 |
| 2DS5 | 0.73 | 0.67 | 0.33 | 0.24 | 0.01 | 0.01 | 0.33 | 0.24 | - |
|  | *p*-values | | | | | | | | |
|  | |  |  |  |  |  |  |  |  |

**b. Linkage disequilibrium analyses between pairs of *KIR* genes in ‘unrelated sample’ of Lanoh**

| KIR Genes | 2DL1 | 2DL3 | 2DL2 | 2DL5 | 3DS1 | 2DS1 | 2DS2 | 2DS3 | 2DS5 |
| --- | --- | --- | --- | --- | --- | --- | --- | --- | --- |
|  | Correlation Matrix (Pearson) | | | | | | | | |
| 2DL1 | - | 0.53 | -0.13 | 0.33 | 0.19 | 0.19 | -0.13 | 0.33 | 0.13 |
| 2DL3 | 0.04 | - | -0.25 | -0.07 | 0.00 | 0.00 | -0.25 | -0.07 | -0.17 |
| 2DL2 | 0.63 | 0.37 | - | 0.27 | 0.00 | 0.00 | 1.00 | 0.27 | 0.25 |
| 2DL5 | 0.23 | 0.81 | 0.33 | - | 0.58 | 0.58 | 0.27 | 1.00 | 0.41 |
| 3DS1 | 0.50 | 1.00 | 1.00 | 0.02 | - | 1.00 | 0.00 | 0.58 | 0.71 |
| 2DS1 | 0.50 | 1.00 | 1.00 | 0.02 | < 0.01 | - | 0.00 | 0.58 | 0.71 |
| 2DS2 | 0.63 | 0.37 | < 0.01 | 0.33 | 1.00 | 1.00 | - | 0.27 | 0.25 |
| 2DS3 | 0.23 | 0.81 | 0.33 | < 0.01 | 0.02 | 0.02 | 0.33 | - | 0.41 |
| 2DS5 | 0.63 | 0.55 | 0.37 | 0.13 | < 0.01 | < 0.01 | 0.37 | 0.13 | - |
|  | *p*-values | | | | | | | | |

**c. Linkage disequilibrium analyses between pairs of *KIR* genes in ‘total sample’ of Batek**

| KIR Genes | 3DL1 | 2DL3 | 2DS4 | 2DL5 | 3DS1 | 2DS1 | 2DS3 | 2DS5 |
| --- | --- | --- | --- | --- | --- | --- | --- | --- |
|  | Correlation Matrix (Pearson) | | | | | | | |
| 3DL1 | - | -0.26 | 0.78 | -0.08 | -0.17 | -0.17 | -0.08 | 0.13 |
| 2DL3 | 0.20 | - | -0.06 | -0.22 | 0.37 | 0.37 | -0.22 | -0.29 |
| 2DS4 | < 0.01 | 0.77 | - | -0.10 | -0.22 | -0.22 | -0.10 | -0.10 |
| 2DL5 | 0.69 | 0.27 | 0.60 | - | -0.08 | -0.08 | -0.04 | 0.16 |
| 3DS1 | 0.39 | 0.06 | 0.26 | 0.69 | - | 1.00 | -0.08 | 0.35 |
| 2DS1 | 0.39 | 0.06 | 0.26 | 0.69 | < 0.01 | - | -0.08 | 0.35 |
| 2DS3 | 0.69 | 0.27 | 0.60 | 0.85 | 0.69 | 0.69 | - | 0.16 |
| 2DS5 | 0.51 | 0.15 | 0.62 | 0.42 | 0.08 | 0.08 | 0.42 | - |
|  | *p*-values | | | | | | | |

**d. Linkage disequilibrium analyses between pairs of *KIR* genes in ‘unrelated sample’ of Batek**

| KIR Genes | 3DL1 | 2DL3 | 2DS4 | 2DL5 | 3DS1 | 2DS1 | 2DS3 | 2DS5 |
| --- | --- | --- | --- | --- | --- | --- | --- | --- |
|  | Correlation Matrix (Pearson) | | | | | | | |
| 3DL1 | - | -0.17 | 1.00 | -0.10 | -0.19 | -0.19 | -0.10 | -0.02 |
| 2DL3 | 0.49 | - | -0.17 | -0.25 | 0.41 | 0.41 | -0.25 | -0.19 |
| 2DS4 | < 0.01 | 0.49 | - | -0.10 | -0.19 | -0.19 | -0.10 | -0.02 |
| 2DL5 | 0.68 | 0.31 | 0.68 | - | -0.10 | -0.10 | -0.06 | 0.16 |
| 3DS1 | 0.44 | 0.08 | 0.44 | 0.68 | - | 1.00 | -0.10 | 0.29 |
| 2DS1 | 0.44 | 0.08 | 0.44 | 0.68 | < 0.01 | - | -0.10 | 0.29 |
| 2DS3 | 0.68 | 0.31 | 0.68 | 0.82 | 0.68 | 0.68 | - | 0.16 |
| 2DS5 | 0.95 | 0.43 | 0.95 | 0.51 | 0.22 | 0.22 | 0.51 | - |
|  | *p*-values | | | | | | | |

**e. Linkage disequilibrium analyses between pairs of *KIR* genes in ‘total sample’ of Kensiu**

| KIR Genes | 3DL1 | 2DL3 | 2DS4 | 2DL2 | 2DL5 | 3DS1 | 2DS1 | 2DS2 | 2DS3 | 2DS5 |
| --- | --- | --- | --- | --- | --- | --- | --- | --- | --- | --- |
|  | Correlation Matrix (Pearson) | | | | | | | | | |
| 3DL1 | - | 0.09 | 1.00 | -0.19 | -0.15 | -0.31 | -0.31 | -0.19 | -0.20 | -0.31 |
| 2DL3 | 0.60 | - | 0.09 | -0.33 | -0.27 | 0.10 | -0.03 | -0.33 | -0.35 | -0.03 |
| 2DS4 | < 0.01 | 0.60 | - | -0.19 | -0.15 | -0.31 | -0.31 | -0.19 | -0.20 | -0.31 |
| 2DL2 | 0.26 | 0.04 | 0.26 | - | 0.81 | 0.14 | 0.26 | 1.00 | 0.94 | 0.26 |
| 2DL5 | 0.36 | 0.11 | 0.36 | < 0.01 | - | 0.36 | 0.49 | 0.81 | 0.76 | 0.49 |
| 3DS1 | 0.06 | 0.54 | 0.06 | 0.40 | 0.03 | - | 0.89 | 0.14 | 0.19 | 0.89 |
| 2DS1 | 0.06 | 0.87 | 0.06 | 0.12 | 0.00 | < 0.01 | - | 0.26 | 0.30 | 1.00 |
| 2DS2 | 0.26 | 0.04 | 0.26 | < 0.01 | < 0.01 | 0.40 | 0.12 | - | 0.94 | 0.26 |
| 2DS3 | 0.23 | 0.03 | 0.23 | < 0.01 | < 0.01 | 0.25 | 0.06 | < 0.01 | - | 0.30 |
| 2DS5 | 0.06 | 0.87 | 0.06 | 0.12 | 0.00 | < 0.01 | < 0.01 | 0.12 | 0.06 | - |
|  | *p*-values | | | | | | | | | |

**f. Linkage disequilibrium analyses between pairs of *KIR* genes in ‘unrelated sample’ of Kensiu**

| KIR Genes | 3DL1 | 2DL3 | 2DS4 | 2DL2 | 2DL5 | 3DS1 | 2DS1 | 2DS2 | 2DS3 | 2DS5 |
| --- | --- | --- | --- | --- | --- | --- | --- | --- | --- | --- |
|  | Correlation Matrix (Pearson) | | | | | | | | | |
| 3DL1 | - | 0.23 | 1.00 | -0.24 | -0.19 | -0.33 | -0.36 | -0.24 | -0.27 | -0.36 |
| 2DL3 | 0.31 | - | 0.23 | -0.24 | -0.19 | -0.33 | -0.36 | -0.24 | -0.27 | -0.36 |
| 2DS4 | < 0.01 | 0.31 | - | -0.24 | -0.19 | -0.33 | -0.36 | -0.24 | -0.27 | -0.36 |
| 2DL2 | 0.28 | 0.28 | 0.28 | - | 0.77 | 0.11 | 0.26 | 1.00 | 0.90 | 0.26 |
| 2DL5 | 0.40 | 0.40 | 0.40 | < 0.01 | - | 0.33 | 0.52 | 0.77 | 0.69 | 0.52 |
| 3DS1 | 0.13 | 0.13 | 0.13 | 0.62 | 0.14 | - | 0.91 | 0.11 | 0.23 | 0.91 |
| 2DS1 | 0.10 | 0.10 | 0.10 | 0.24 | 0.01 | < 0.01 | - | 0.26 | 0.36 | 1.00 |
| 2DS2 | 0.28 | 0.28 | 0.28 | < 0.01 | < 0.01 | 0.62 | 0.24 | - | 0.90 | 0.26 |
| 2DS3 | 0.22 | 0.22 | 0.22 | < 0.01 | 0.00 | 0.31 | 0.10 | < 0.01 | - | 0.36 |
| 2DS5 | 0.10 | 0.10 | 0.10 | 0.24 | 0.01 | < 0.01 | < 0.01 | 0.24 | 0.10 | - |
|  | *p*-values | | | | | | | | | |

**g. Linkage disequilibrium analyses between pairs of *KIR* genes in ‘total sample’ of Che Wong**

| KIR Genes | 3DL1 | 2DL3 | 2DS4 | 2DL2 | 2DL5 | 3DS1 | 2DS1 | 2DS2 | 2DS3 | 2DS5 |
| --- | --- | --- | --- | --- | --- | --- | --- | --- | --- | --- |
|  | Correlation Matrix (Pearson) | | | | | | | | | |
| 3DL1 | - | 0.47 | -0.04 | -0.18 | -0.11 | -0.19 | -0.21 | -0.18 | -0.17 | 0.10 |
| 2DL3 | 0.01 | - | -0.08 | -0.38 | -0.24 | 0.00 | -0.03 | -0.38 | -0.35 | -0.04 |
| 2DS4 | 0.85 | 0.69 | - | 0.21 | -0.11 | -0.19 | -0.21 | 0.21 | -0.17 | 0.10 |
| 2DL2 | 0.36 | 0.05 | 0.29 | - | 0.45 | -0.07 | 0.01 | 1.00 | 0.50 | -0.04 |
| 2DL5 | 0.57 | 0.23 | 0.57 | 0.02 | - | 0.58 | 0.54 | 0.45 | 0.67 | 0.30 |
| 3DS1 | 0.33 | 1.00 | 0.33 | 0.72 | 0.00 | - | 0.93 | -0.07 | 0.14 | 0.52 |
| 2DS1 | 0.29 | 0.88 | 0.29 | 0.98 | 0.00 | < 0.01 | - | 0.01 | 0.23 | 0.39 |
| 2DS2 | 0.36 | 0.05 | 0.29 | < 0.01 | 0.02 | 0.72 | 0.98 | - | 0.50 | -0.04 |
| 2DS3 | 0.40 | 0.06 | 0.40 | 0.01 | 0.00 | 0.46 | 0.24 | 0.01 | - | -0.43 |
| 2DS5 | 0.61 | 0.86 | 0.61 | 0.85 | 0.12 | 0.00 | 0.04 | 0.85 | 0.02 | - |
|  | *p*-values | | | | | | | | | |

**h. Linkage disequilibrium analyses between pairs of *KIR* genes in ‘unrelated sample’ of Che Wong**

| KIR Genes | 3DL1 | 2DL3 | 2DL2 | 2DL5 | 3DS1 | 2DS1 | 2DS2 | 2DS3 | 2DS5 |
| --- | --- | --- | --- | --- | --- | --- | --- | --- | --- |
|  | Correlation Matrix (Pearson) | | | | | | | | |
| 3DL1 | - | 0.68 | -0.20 | -0.10 | -0.20 | -0.23 | -0.20 | -0.23 | 0.17 |
| 2DL3 | 0.00 | - | -0.29 | -0.14 | 0.10 | 0.05 | -0.29 | -0.33 | 0.25 |
| 2DL2 | 0.46 | 0.27 | - | 0.10 | -0.33 | -0.16 | 1.00 | 0.36 | -0.31 |
| 2DL5 | 0.72 | 0.60 | 0.72 | - | 0.49 | 0.43 | 0.10 | 0.43 | 0.25 |
| 3DS1 | 0.46 | 0.72 | 0.21 | 0.06 | - | 0.88 | -0.33 | -0.16 | 0.52 |
| 2DS1 | 0.40 | 0.86 | 0.55 | 0.10 | < 0.01 | - | -0.16 | -0.02 | 0.32 |
| 2DS2 | 0.46 | 0.27 | < 0.01 | 0.72 | 0.21 | 0.55 | - | 0.36 | -0.31 |
| 2DS3 | 0.40 | 0.21 | 0.17 | 0.10 | 0.55 | 0.95 | 0.17 | - | -0.76 |
| 2DS5 | 0.52 | 0.34 | 0.24 | 0.34 | 0.04 | 0.22 | 0.24 | < 0.01 | - |
|  | *p*-values | | | | | | | | |

**i. Linkage disequilibrium analyses between pairs of *KIR* genes in ‘total sample’ of Semai**

| KIR Genes | 2DL1 | 2DL3 | 2DL2 | 2DL5 | 3DS1 | 2DS1 | 2DS2 | 2DS3 | 2DS5 |
| --- | --- | --- | --- | --- | --- | --- | --- | --- | --- |
|  | Correlation Matrix (Pearson) | | | | | | | | |
| 2DL1 | - | 0.42 | -0.18 | 0.09 | 0.05 | 0.05 | -0.16 | 0.09 | 0.05 |
| 2DL3 | 0.01 | - | -0.43 | -0.51 | -0.17 | -0.17 | -0.38 | -0.56 | -0.17 |
| 2DL2 | 0.28 | 0.01 | - | 0.24 | 0.12 | 0.12 | 0.90 | 0.31 | 0.12 |
| 2DL5 | 0.58 | 0.00 | 0.16 | - | 0.52 | 0.52 | 0.43 | 0.93 | 0.52 |
| 3DS1 | 0.77 | 0.31 | 0.47 | 0.00 | - | 1.00 | 0.09 | 0.33 | 1.00 |
| 2DS1 | 0.77 | 0.31 | 0.47 | < 0.01 | < 0.01 | - | 0.09 | 0.33 | 1.00 |
| 2DS2 | 0.34 | 0.02 | < 0.01 | 0.01 | 0.59 | 0.59 | - | 0.51 | 0.09 |
| 2DS3 | 0.61 | 0.00 | 0.07 | < 0.01 | 0.05 | 0.05 | 0.00 | - | 0.33 |
| 2DS5 | 0.77 | 0.31 | 0.47 | 0.00 | < 0.01 | < 0.01 | 0.59 | 0.05 | - |
|  | *p*-values | | | | | | | | |

**j. Linkage disequilibrium analyses between pairs of *KIR* genes in ‘unrelated sample’ of Semai**

| KIR Genes | 2DL3 | 2DL2 | 2DL5 | 3DS1 | 2DS1 | 2DS2 | 2DS3 | 2DS5 |
| --- | --- | --- | --- | --- | --- | --- | --- | --- |
|  | Correlation Matrix (Pearson) | | | | | | | |
| 2DL3 | - | -0.38 | -0.55 | 0.09 | 0.09 | -0.33 | -0.60 | 0.09 |
| 2DL2 | 0.04 | - | 0.22 | 0.03 | 0.03 | 0.87 | 0.30 | 0.03 |
| 2DL5 | 0.00 | 0.25 | - | 0.44 | 0.44 | 0.44 | 0.91 | 0.44 |
| 3DS1 | 0.63 | 0.88 | 0.02 | - | 1.00 | -0.01 | 0.16 | 1.00 |
| 2DS1 | 0.63 | 0.88 | 0.02 | < 0.01 | - | -0.01 | 0.16 | 1.00 |
| 2DS2 | 0.08 | < 0.01 | 0.02 | 0.96 | 0.96 | - | 0.54 | -0.01 |
| 2DS3 | < 0.01 | 0.11 | < 0.01 | 0.39 | 0.39 | 0.00 | - | 0.16 |
| 2DS5 | 0.63 | 0.88 | 0.02 | < 0.01 | < 0.01 | 0.96 | 0.39 | - |
|  | *p*-values | | | | | | | |

**k. Linkage disequilibrium analyses between pairs of *KIR* genes in ‘total sample’ of Orang Kanaq**

| KIR Genes | 3DL1 | 2DS4 | 2DL5 | 3DS1 | 2DS1 | 2DS5 |
| --- | --- | --- | --- | --- | --- | --- |
|  | Correlation Matrix (Pearson) | | | | | |
| 3DL1 | - | 1.00 | -0.10 | -0.10 | -0.10 | -0.15 |
| 2DS4 | < 0.01 | - | -0.10 | -0.10 | -0.10 | -0.15 |
| 2DL5 | 0.77 | 0.77 | - | 1.00 | 1.00 | 0.67 |
| 3DS1 | 0.77 | 0.77 | < 0.01 | - | 1.00 | 0.67 |
| 2DS1 | 0.77 | 0.77 | < 0.01 | < 0.01 | - | 0.67 |
| 2DS5 | 0.66 | 0.66 | 0.02 | 0.02 | 0.02 | - |
|  | *p*-values | | | | | |

**l. Linkage disequilibrium analyses between pairs of *KIR* genes in ‘unrelated sample’ of Orang Kanaq**

| KIR Genes | 3DL1 | 2DS4 | 2DS3 | 2DS5 |
| --- | --- | --- | --- | --- |
|  | Correlation Matrix (Pearson) | | | |
| 3DL1 | - | 1.00 | 0.17 | -0.17 |
| 2DS4 | < 0.01 | - | 0.17 | -0.17 |
| 2DS3 | 0.72 | 0.72 | - | -1.00 |
| 2DS5 | 0.72 | 0.72 | < 0.01 | - |
|  | *p*-values | | | |
