# Supplementary material for: Ancient Genetic Signatures of Orang Asli Revealed by Killer Immunoglobulin-Like Receptor Gene Polymorphisms
Source: PLoS One. 2015 Nov 13;10(11):e0141536. doi: 10.1371/journal.pone.0141536 (PMC4643969; doi:10.1371/journal.pone.0141536)
Supplement: S4 Table — (DOC) [file pone.0141536.s004.doc]

**S4 Table. Homogeneity tests (*p*-values) between pairs of OA subgroups using (a) *KIR3DL1* and *KIR2DL1*, (b) *KIR2DL3* and *KIR2DS4*, (c) *KIR2DL2* and *KIR2DL5*, (d) *KIR3DS1* and *KIR2DS1*, (e) *KIR2DS2* and *KIR2DS3* and (f) *KIR2DS5* with *p*-values <0.05 as level of significance.** C.C.,cannot be calculated.

**a.**

| Orang Asli subgroups | | *KIR3DL1* | | | | | | | | | | | |
| --- | --- | --- | --- | --- | --- | --- | --- | --- | --- | --- | --- | --- | --- |
|  |  | Lanoh | | Batek | | Kensiu | | Che Wong | | Semai | | Orang Kanaq | |
|  |  | Total | Unrelated | Total | Unrelated | Total | Unrelated | Total | Unrelated | Total | Unrelated | Total | Unrelated |
| Lanoh | Total | ~ |  | 0.13 |  | 0.39 |  | 0.33 |  | C.C. |  | 0.65 |  |
|  | Unrelated |  | ~ |  | 0.32 |  | 0.38 |  | 0.33 |  | C.C. |  | 0.69 |
| Batek | Total | 0.98 |  | ~ |  | 0.63 |  | 0.33 |  | 0.06 |  | 0.64 |  |
|  | Unrelated |  | 0.90 |  | ~ |  | 0.85 |  | 0.73 |  | 0.11 |  | 0.92 |
| Kensiu | Total | 0.85 |  | C.C. |  | ~ |  | 0.84 |  | 0.25 |  | 0.90 |  |
|  | Unrelated |  | 0.85 |  | C.C. |  | ~ |  | 0.84 |  | 0.15 |  | 0.97 |
| Che Wong | Total | 0.97 |  | C.C. |  | C.C. |  | ~ |  | 0.89 |  | 0.48 |  |
|  | Unrelated |  | 0.97 |  | C.C. |  | C.C. |  | ~ |  | 0.76 |  | 0.53 |
| Semai | Total | 0.80 |  | 0.39 |  | 0.99 |  | 0.38 |  | ~ |  | 0.51 |  |
|  | Unrelated |  | 0.73 |  | C.C. |  | C.C. |  | C.C. |  | ~ |  | 0.43 |
| Orang Kanaq | Total | 0.51 |  | C.C. |  | C.C. |  | C.C. |  | 0.58 |  | ~ |  |
|  | Unrelated |  | 0.48 |  | C.C. |  | C.C. |  | C.C. |  | C.C. |  | ~ |
|  |  | *KIR2DL1* | | | | | | | | | | | |

**b.**

| Orang Asli subgroups | | *KIR2DL3* | | | | | | | | | | | |
| --- | --- | --- | --- | --- | --- | --- | --- | --- | --- | --- | --- | --- | --- |
|  |  | Lanoh | | Batek | | Kensiu | | Che Wong | | Semai | | Orang Kanaq | |
|  |  | Total | Unrelated | Total | Unrelated | Total | Unrelated | Total | Unrelated | Total | Unrelated | Total | Unrelated |
| Lanoh | Total | ~ |  | 0.03 |  | 0.85 |  | 0.63 |  | 0.52 |  | 0.21 |  |
|  | Unrelated |  | ~ |  | 0.11 |  | 0.95 |  | 0.94 |  | 0.67 |  | 0.54 |
| Batek | Total | 0.03 |  | ~ |  | 0.01 |  | <0.01 |  | <0.01 |  | <0.01 |  |
|  | Unrelated |  | 0.32 |  | ~ |  | 0.02 |  | 0.03 |  | <0.01 |  | 0.05 |
| Kensiu | Total | 0.39 |  | 0.20 |  | ~ |  | 0.70 |  | 0.58 |  | 0.23 |  |
|  | Unrelated |  | 0.38 |  | 0.85 |  | ~ |  | 0.92 |  | 0.72 |  | 0.75 |
| Che Wong | Total | 0.33 |  | 0.09 |  | 0.84 |  | ~ |  | 0.93 |  | 0.46 |  |
|  | Unrelated |  | C.C. |  | 0.29 |  | 0.35 |  | ~ |  | 0.83 |  | 0.86 |
| Semai | Total | C.C. |  | 0.01 |  | 0.25 |  | 0.89 |  | ~ |  | 0.47 |  |
|  | Unrelated |  | C.C. |  | 0.11 |  | 0.15 |  | C.C. |  | ~ |  | 0.90 |
| Orang Kanaq | Total | 0.65 |  | 0.63 |  | 0.90 |  | 0.48 |  | 0.51 |  | ~ |  |
|  | Unrelated |  | 0.69 |  | 0.92 |  | 0.97 |  | 0.66 |  | 0.43 |  | ~ |
|  |  | *KIR2DS4* | | | | | | | | | | | |

**c.**

| Orang Asli subgroups | | *KIR2DL2* | | | | | | | | | | | |
| --- | --- | --- | --- | --- | --- | --- | --- | --- | --- | --- | --- | --- | --- |
|  |  | Lanoh | | Batek | | Kensiu | | Che Wong | | Semai | | Orang Kanaq | |
|  |  | Total | Unrelated | Total | Unrelated | Total | Unrelated | Total | Unrelated | Total | Unrelated | Total | Unrelated |
| Lanoh | Total | ~ |  | 0.03 |  | 0.86 |  | 0.13 |  | 0.03 |  | <0.01 |  |
|  | Unrelated |  | ~ |  | 0.15 |  | 0.91 |  | 0.50 |  | 0.06 |  | <0.01 |
| Batek | Total | 0.02 |  | ~ |  | 0.01 |  | <0.01 |  | <0.01 |  | <0.01 |  |
|  | Unrelated |  | 0.04 |  | ~ |  | 0.04 |  | 0.01 |  | <0.01 |  | <0.01 |
| Kensiu | Total | 0.56 |  | 0.10 |  | ~ |  | 0.13 |  | 0.02 |  | <0.01 |  |
|  | Unrelated |  | 0.28 |  | 0.43 |  | ~ |  | 0.75 |  | 0.09 |  | <0.01 |
| Che Wong | Total | 0.87 |  | 0.06 |  | 0.94 |  | ~ |  | 0.72 |  | 0.01 |  |
|  | Unrelated |  | 0.18 |  | 0.88 |  | 0.98 |  | ~ |  | 0.41 |  | 0.02 |
| Semai | Total | 0.00 |  | <0.01 |  | <0.01 |  | <0.01 |  | ~ |  | 0.01 |  |
|  | Unrelated |  | 0.08 |  | <0.01 |  | <0.01 |  | 0.98 |  | ~ |  | 0.08 |
| Orang Kanaq | Total | 0.32 |  | 0.50 |  | 0.65 |  | 0.51 |  | <0.01 |  | ~ |  |
|  | Unrelated |  | 0.15 |  | 0.54 |  | 0.56 |  | 0.56 |  | <0.01 |  | ~ |
|  |  | *KIR2DL5* | | | | | | | | | | | |

**d.**

| Orang Asli subgroups | | *KIR3DS1* | | | | | | | | | | | |
| --- | --- | --- | --- | --- | --- | --- | --- | --- | --- | --- | --- | --- | --- |
|  |  | Lanoh | | Batek | | Kensiu | | Che Wong | | Semai | | Orang Kanaq | |
|  |  | Total | Unrelated | Total | Unrelated | Total | Unrelated | Total | Unrelated | Total | Unrelated | Total | Unrelated |
| Lanoh | Total | ~ |  | <0.01 |  | 0.45 |  | 0.39 |  | 0.02 |  | 0.01 |  |
|  | Unrelated |  | ~ |  | 0.01 |  | 0.23 |  | 0.21 |  | 0.07 |  | 0.01 |
| Batek | Total | <0.01 |  | ~ |  | <0.01 |  | 0.01 |  | <0.01 |  | 0.64 |  |
|  | Unrelated |  | 0.01 |  | ~ |  | 0.16 |  | 0.28 |  | <0.01 |  | 0.67 |
| Kensiu | Total | 0.45 |  | <0.01 |  | ~ |  | 0.83 |  | <0.01 |  | 0.03 |  |
|  | Unrelated |  | 0.35 |  | 0.09 |  | ~ |  | 0.83 |  | <0.01 |  | 0.12 |
| Che Wong | Total | 0.54 |  | 0.01 |  | 0.94 |  | ~ |  | <0.01 |  | 0.05 |  |
|  | Unrelated |  | 0.36 |  | 0.15 |  | 0.92 |  | ~ |  | <0.01 |  | 0.17 |
| Semai | Total | 0.02 |  | <0.01 |  | <0.01 |  | <0.01 |  | ~ |  | <0.01 |  |
|  | Unrelated |  | 0.07 |  | <0.01 |  | <0.01 |  | <0.01 |  | ~ |  | <0.01 |
| Orang Kanaq | Total | 0.01 |  | 0.64 |  | 0.03 |  | 0.03 |  | <0.01 |  | ~ |  |
|  | Unrelated |  | 0.01 |  | 0.67 |  | 0.08 |  | 0.11 |  | <0.01 |  | ~ |
|  |  | *KIR2DS1* | | | | | | | | | | | |

**e.**

| Orang Asli subgroups | | *KIR2DS2* | | | | | | | | | | | |
| --- | --- | --- | --- | --- | --- | --- | --- | --- | --- | --- | --- | --- | --- |
|  |  | Lanoh | | Batek | | Kensiu | | Che Wong | | Semai | | Orang Kanaq | |
|  |  | Total | Unrelated | Total | Unrelated | Total | Unrelated | Total | Unrelated | Total | Unrelated | Total | Unrelated |
| Lanoh | Total | ~ |  | 0.03 |  | 0.81 |  | 0.13 |  | 0.07 |  | <0.01 |  |
|  | Unrelated |  | ~ |  | 0.15 |  | 0.91 |  | 0.50 |  | 0.13 |  | <0.01 |
| Batek | Total | 0.02 |  | ~ |  | 0.01 |  | <0.01 |  | <0.01 |  | <0.01 |  |
|  | Unrelated |  | 0.04 |  | ~ |  | 0.04 |  | 0.01 |  | <0.01 |  | <0.01 |
| Kensiu | Total | 0.95 |  | 0.01 |  | ~ |  | 0.23 |  | 0.13 |  | <0.01 |  |
|  | Unrelated |  | 0.87 |  | 0.08 |  | ~ |  | 0.75 |  | 0.22 |  | <0.01 |
| Che Wong | Total | 0.52 |  | <0.01 |  | 0.49 |  | ~ |  | 0.86 |  | 0.01 |  |
|  | Unrelated |  | 0.83 |  | 0.02 |  | 0.68 |  | ~ |  | 0.70 |  | 0.02 |
| Semai | Total | <0.01 |  | <0.01 |  | <0.01 |  | 0.01 |  | ~ |  | 0.01 |  |
|  | Unrelated |  | 0.04 |  | <0.01 |  | <0.01 |  | 0.07 |  | ~ |  | 0.04 |
| Orang Kanaq | Total | <0.01 |  | <0.01 |  | <0.01 |  | 0.02 |  | 0.62 |  | ~ |  |
|  | Unrelated |  | 0.12 |  | <0.01 |  | 0.04 |  | 0.16 |  | 0.96 |  | ~ |
|  |  | *KIR2DS3* | | | | | | | | | | | |

**f.**

| Orang Asli subgroups | | *KIR2DS5* | | | | | | | | | | | |
| --- | --- | --- | --- | --- | --- | --- | --- | --- | --- | --- | --- | --- | --- |
|  |  | Lanoh | | Batek | | Kensiu | | Che Wong | | Semai | | Orang Kanaq | |
|  |  | Total | Unrelated | Total | Unrelated | Total | Unrelated | Total | Unrelated | Total | Unrelated | Total | Unrelated |
| Lanoh | Total | ~ |  | 0.04 |  | 0.01 |  | 0.54 |  | 0.98 |  | <0.01 |  |
|  | Unrelated |  | ~ |  | 0.71 |  | 0.08 |  | 0.76 |  | 0.43 |  | 0.01 |
| Batek | Total |  |  | ~ |  | 0.78 |  | 0.21 |  | <0.01 |  | 0.05 |  |
|  | Unrelated |  |  |  | ~ |  | 0.25 |  | 0.98 |  | 0.06 |  | 0.04 |
| Kensiu | Total |  |  |  |  | ~ |  | 0.06 |  | <0.01 |  | 0.09 |  |
|  | Unrelated |  |  |  |  |  | ~ |  | 0.27 |  | <0.01 |  | 0.30 |
| Che Wong | Total |  |  |  |  |  |  | ~ |  | 0.24 |  | <0.01 |  |
|  | Unrelated |  |  |  |  |  |  |  | ~ |  | 0.08 |  | 0.05 |
| Semai | Total |  |  |  |  |  |  |  |  | ~ |  | <0.01 |  |
|  | Unrelated |  |  |  |  |  |  |  |  |  | ~ |  | <0.01 |
| Orang Kanaq | Total |  |  |  |  |  |  |  |  |  |  | ~ |  |
|  | Unrelated |  |  |  |  |  |  |  |  |  |  |  | ~ |
