# Supplementary material for: Ancient Genetic Signatures of Orang Asli Revealed by Killer Immunoglobulin-Like Receptor Gene Polymorphisms
Source: PLoS One. 2015 Nov 13;10(11):e0141536. doi: 10.1371/journal.pone.0141536 (PMC4643969; doi:10.1371/journal.pone.0141536)
Supplement: S5 Table — (DOC) [file pone.0141536.s005.doc]

**S5 Table. *KIR* estimated genes (*gF*) and haplotypes frequencies for global populations.**

| Populations | KIR Genes | | | | | | | | | | | Haplotype A | Haplotype B | References |
| --- | --- | --- | --- | --- | --- | --- | --- | --- | --- | --- | --- | --- | --- | --- |
|  | 3DL1 | 2DL1 | 2DL3 | 2DS4 | 2DL2 | 2DL5 | 3DS1 | 2DS1 | 2DS2 | 2DS3 | 2DS5 | (*F*) | (*F*) |  |
| Lanoh | 1.00 | 0.80 | 0.52 | 1.00 | 0.52 | 0.44 | 0.19 | 0.19 | 0.52 | 0.44 | 0.06 | 0.44 | 0.56 | present study |
| Batek | 0.61 | 1.00 | 0.25 | 0.53 | 1.00 | 0.80 | 0.61 | 0.61 | 1.00 | 0.80 | 0.23 | 0.17 | 0.83 | present study |
| Kensiu | 0.70 | 1.00 | 0.54 | 0.70 | 0.43 | 0.51 | 0.27 | 0.27 | 0.43 | 0.41 | 0.27 | 0.46 | 0.54 | present study |
| Che Wong | 0.80 | 1.00 | 0.63 | 0.80 | 0.32 | 0.50 | 0.29 | 0.27 | 0.32 | 0.34 | 0.11 | 0.52 | 0.48 | present study |
| Semai | 1.00 | 0.83 | 0.63 | 1.00 | 0.27 | 0.13 | 0.04 | 0.04 | 0.30 | 0.12 | 0.04 | 0.66 | 0.34 | present study |
| Orang Kanaq | 0.70 | 1.00 | 1.00 | 0.70 | 0.00 | 0.70 | 0.70 | 0.70 | 0.00 | 0.05 | 0.58 | 0.50 | 0.50 | present study |
| Ghana | 1.00 | 0.74 | 0.61 | 0.86 | 0.32 | 0.34 | 0.03 | 0.03 | 0.34 | 0.19 | 0.12 | 0.57 | 0.43 | [15] |
| Japan | 0.90 | 1.00 | 1.00 | 0.90 | 0.06 | 0.20 | 0.18 | 0.18 | 0.06 | 0.07 | 0.12 | 0.79 | 0.21 | [18] |
| South Africa Xhosa | 1.00 | 0.76 | 0.42 | 1.00 | 0.45 | 0.60 | 0.02 | 0.06 | 0.40 | 0.21 | 0.40 | 0.38 | 0.62 | [19] |
| South Africa San | 0.90 | 0.80 | 0.37 | 1.00 | 0.50 | 0.44 | 0.01 | 0.08 | 0.49 | 0.13 | 0.41 | 0.39 | 0.63 | [20] |
| India Paravar | 0.60 | 0.83 | 0.54 | 0.60 | 0.44 | 0.59 | 0.37 | 0.41 | 0.50 | 0.29 | 0.42 | 0.35 | 0.65 | [21] |
| India North | 0.65 | 0.65 | 0.41 | 0.56 | 0.54 | 0.54 | 0.22 | 0.32 | 0.39 | 0.25 | 0.29 | 0.29 | 0.72 | [22] |
| Vietnam | 0.65 | 0.86 | 0.86 | 0.65 | 0.20 | N.T | 0.23 | 0.21 | 0.23 | 0.18 | N.T | 0.79 | 0.21 | [23] |
| Australian Aborigine | 0.33 | 0.47 | 0.43 | 0.31 | 0.54 | N.T | 0.53 | 0.58 | 0.60 | 0.56 | 0.00 | 0.21 | 0.79 | [23] |
| Borneo Kalimantan | 0.80 | 0.59 | 1.00 | 0.80 | 0.18 | 0.17 | 0.13 | 0.11 | 0.17 | 0.11 | 0.05 | 0.71 | 0.29 | [24] |
| East Timor | 0.49 | 0.80 | 0.76 | 0.68 | 0.29 | 0.51 | 0.42 | 0.32 | 0.25 | 0.23 | 0.37 | 0.44 | 0.56 | [24] |
| Indonesia Java | 0.74 | 0.64 | 1.00 | 0.86 | 0.21 | 0.34 | 0.30 | 0.35 | 0.20 | 0.17 | 0.17 | 0.58 | 0.42 | [24] |
| Papuan Irian Jaya | 0.27 | 0.63 | 0.47 | 0.49 | 0.43 | 0.63 | 0.47 | 0.49 | 0.40 | 0.42 | 0.38 | 0.22 | 0.78 | [24] |
| Solomon | 0.48 | 0.86 | 0.53 | 0.48 | 0.45 | 0.55 | 0.31 | 0.41 | 0.43 | 0.39 | 0.23 | 0.41 | 0.59 | [36] |
| Kelantan Malays | 0.64 | 0.74 | 0.59 | 0.39 | 0.18 | 0.34 | 0.29 | 0.27 | 0.18 | 0.15 | 0.25 | 0.48 | 0.52 | [47] |
| Pattani Malays | 1.00 | 1.00 | 0.68 | 0.68 | 0.31 | 0.23 | 0.16 | 0.18 | 0.31 | 0.12 | 0.12 | 0.60 | 0.40 | [47] |
| Banjar Malays | 0.83 | 0.83 | 0.74 | 0.43 | 0.29 | 0.37 | 0.25 | 0.21 | 0.29 | 0.21 | 0.18 | 0.52 | 0.48 | [47] |
| Jawa Malays | 0.83 | 0.83 | 0.74 | 0.55 | 0.21 | 0.23 | 0.15 | 0.15 | 0.21 | 0.11 | 0.09 | 0.65 | 0.35 | [47] |

**S2 Table**. Continued.

| Populations | KIR Genes | | | | | | | | | | | Haplotype A | Haplotype B | References |
| --- | --- | --- | --- | --- | --- | --- | --- | --- | --- | --- | --- | --- | --- | --- |
|  | 3DL1 | 2DL1 | 2DL3 | 2DS4 | 2DL2 | 2DL5 | 3DS1 | 2DS1 | 2DS2 | 2DS3 | 2DS5 | (*F*) | (*F*) |  |
| Biaka Pygmies | 1.00 | 1.00 | 0.68 | 1.00 | 0.38 | 0.51 | 0.04 | 0.02 | 0.36 | 0.09 | 0.42 | C.C. | C.C. | [49] |
| Mandenka | 1.00 | 1.00 | 0.70 | 0.80 | 0.45 | 0.34 | 0.09 | 0.12 | 0.31 | 0.22 | 0.16 | C.C. | C.C. | [49] |
| Mozabite | 1.00 | 1.00 | 0.67 | 1.00 | 0.39 | 0.18 | 0.04 | 0.04 | 0.45 | 0.18 | 0.04 | C.C. | C.C. | [49] |
| Pathan | 0.63 | 0.78 | 0.56 | 0.70 | 0.43 | 0.56 | 0.31 | 0.38 | 0.51 | 0.28 | 0.38 | C.C. | C.C. | [49] |
| Barusho | 0.78 | 0.68 | 0.50 | 0.78 | 0.50 | 0.50 | 0.26 | 0.41 | 0.50 | 0.29 | 0.29 | C.C. | C.C. | [49] |
| NAN Melanesia | 0.31 | 0.78 | 0.46 | 0.43 | 0.70 | 1.00 | 0.63 | 0.70 | 0.56 | 0.78 | 0.21 | C.C. | C.C. | [49] |
| Sulawesi Minahasan | 0.50 | 0.78 | 0.74 | 0.72 | 0.31 | 0.45 | 0.43 | 0.41 | 0.31 | 0.33 | 0.28 | 0.44 | 0.56 | [50] |
| Thailand North East | 0.78 | 0.78 | 0.68 | 0.76 | 0.23 | 0.31 | 0.25 | 0.27 | 0.23 | 0.18 | 0.16 | 0.60 | 0.40 | [51] |
| Taiwan Han | 1.00 | 1.00 | 1.00 | 1.00 | 0.14 | 0.20 | 0.15 | 0.16 | 0.13 | 0.11 | 0.10 | 0.76 | 0.24 | [52] |
| South Korea | 0.76 | 0.90 | 0.90 | 0.76 | 0.07 | 0.21 | 0.20 | 0.21 | 0.09 | 0.08 | 0.15 | 0.75 | 0.25 | [53] |
| Tokelau | 0.61 | 0.67 | 0.80 | 0.80 | 0.23 | 0.34 | 0.18 | 0.20 | 0.25 | 0.19 | 0.14 | 0.65 | 0.35 | [54] |
| Tonga | 0.60 | 1.00 | 0.76 | 0.48 | 0.30 | 0.31 | 0.19 | 0.16 | 0.29 | 0.19 | 0.22 | 0.54 | 0.46 | [54] |
| Samoa | 0.72 | 0.86 | 0.86 | 0.76 | 0.21 | 0.28 | 0.21 | 0.19 | 0.23 | 0.19 | 0.12 | 0.68 | 0.32 | [54] |
| Venezuela Yucpa | 0.46 | 0.46 | 0.46 | 0.46 | 0.46 | 0.49 | 0.49 | 0.49 | 0.46 | 0.00 | 0.49 | 0.47 | 0.53 | [55] |
| Venezuela Bari | 0.59 | 0.67 | 0.67 | 0.56 | 0.25 | 0.40 | 0.38 | 0.40 | 0.25 | 0.00 | 0.40 | 0.45 | 0.55 | [55] |
| Papua New Guinea Nasioi | 0.36 | 0.70 | 0.36 | N.T | 0.80 | N.T | 0.48 | 0.70 | 0.70 | N.T | N.T | C.C. | C.C. | [56] |
| Cambodia | 0.78 | 0.70 | 0.63 | N.T | 0.26 | N.T | 0.28 | 0.25 | 0.25 | N.T | N.T | C.C. | C.C. | [56] |
| Taiwan Atayal | 1.00 | 1.00 | 1.00 | N.T | 0.00 | N.T | 0.19 | 0.18 | 0.00 | N.T | N.T | C.C. | C.C. | [56] |
| Taiwan Ami | 1.00 | 0.72 | 0.61 | N.T | 0.39 | N.T | 0.18 | 0.21 | 0.37 | N.T | N.T | C.C. | C.C. | [56] |
| Maori | 0.80 | 0.72 | 0.63 | 0.86 | 0.29 | 0.31 | 0.24 | 0.23 | 0.29 | 0.15 | 0.15 | 0.61 | 0.39 | [57] |
| Brazil Kaingang | 0.53 | 0.64 | 0.60 | 0.53 | 0.36 | 0.43 | 0.45 | 0.45 | 0.36 | 0.00 | 0.43 | 0.48 | 0.53 | [58] |
| Brazil Guarani | 0.50 | 0.60 | 0.59 | 0.50 | 0.31 | 0.49 | 0.53 | 0.49 | 0.31 | 0.08 | 0.40 | 0.41 | 0.59 | [58] |
| Argentina Wichis | 0.67 | 0.61 | 0.61 | 0.67 | 0.38 | 0.32 | 0.32 | 0.32 | 0.38 | 0.02 | 0.31 | 0.57 | 0.43 | [59] |

The datasets were used to construct Figs. 2 and 3. C.C = cannot be calculated; *F* = frequency, N.T = not tested.

**References**

**49**. Hollenbach JA, Nocedal I, Ladner MB, Single RM, Trachtenberg EA. Killer cell immunoglobulin-like receptor (KIR) gene content variation in the HGDP-CEPH populations. Immunogenetics. 2012;64: 719-737.

**50**. Velickovic M, Velickovic Z, [Panigoro R](http://www.ncbi.nlm.nih.gov/pubmed/?term=Panigoro R%5BAuthor%5D&cauthor=true&cauthor_uid=20670355), [Dunckley H](http://www.ncbi.nlm.nih.gov/pubmed/?term=Dunckley H%5BAuthor%5D&cauthor=true&cauthor_uid=20670355). Diversity of killer cell immunoglobulin-like receptor genes in Indonesian populations of Sumatra, Sulawesi and Moluccas Islands. Tissue Antigens. 2010;76: 325-330.

**51**. Chaisri S, Kitcharoen K, Romphruk AV, Romphruk A, Witt CS, Leelayuwat C. Polymorphisms of killer immunoglobulin-like receptors (KIRs) and HLA ligands in northeastern Thais. Immunogenetics. 2013;65: 645-653.

**52**. Wu GQ,  Zhao YM, Lai XY, [Yang KL](http://www.ncbi.nlm.nih.gov/pubmed/?term=Yang KL%5BAuthor%5D&cauthor=true&cauthor_uid=19761533), Zhu FM, Zhang W, et al. Distribution of killer-cell immunoglobulin-like receptor genes in eastern mainland Chinese Han and Taiwanese Han populations. Tissue Antigens. 2009;74: 499-507.

**53**. Whang DH, [Park H](http://www.ncbi.nlm.nih.gov/pubmed/?term=Park H%5BAuthor%5D&cauthor=true&cauthor_uid=15695000), [Yoon JA](http://www.ncbi.nlm.nih.gov/pubmed/?term=Yoon JA%5BAuthor%5D&cauthor=true&cauthor_uid=15695000), [Park MH](http://www.ncbi.nlm.nih.gov/pubmed/?term=Park MH%5BAuthor%5D&cauthor=true&cauthor_uid=15695000). Haplotype analysis of killer cell immunoglobulin-like receptor genes in 77 Korean families. Hum Immunol. 2005;66: 146-154.

**54**. Velickovic M, Velickovic Z, Dunckley H. Diversity of killer cell immunoglobulin-like receptor genes in Pacific Islands populations. Immunogenetics. 2006;58: 523-532.

**55**. Gendzekhadze K, [Norman PJ](http://www.ncbi.nlm.nih.gov/pubmed/?term=Norman PJ%5BAuthor%5D&cauthor=true&cauthor_uid=16738943), [Abi-Rached L](http://www.ncbi.nlm.nih.gov/pubmed/?term=Abi-Rached L%5BAuthor%5D&cauthor=true&cauthor_uid=16738943), [Layrisse Z](http://www.ncbi.nlm.nih.gov/pubmed/?term=Layrisse Z%5BAuthor%5D&cauthor=true&cauthor_uid=16738943), [Parham P](http://www.ncbi.nlm.nih.gov/pubmed/?term=Parham P%5BAuthor%5D&cauthor=true&cauthor_uid=16738943). High KIR diversity in Amerindians is maintained using few gene-content haplotypes. Immunogenetics. 2006;58: 474-480.

**56**. Single RM, [Martin MP](http://www.ncbi.nlm.nih.gov/pubmed/?term=Martin MP%5BAuthor%5D&cauthor=true&cauthor_uid=17694058), [Gao X](http://www.ncbi.nlm.nih.gov/pubmed/?term=Gao X%5BAuthor%5D&cauthor=true&cauthor_uid=17694058), [Meyer D](http://www.ncbi.nlm.nih.gov/pubmed/?term=Meyer D%5BAuthor%5D&cauthor=true&cauthor_uid=17694058), [Yeager M](http://www.ncbi.nlm.nih.gov/pubmed/?term=Yeager M%5BAuthor%5D&cauthor=true&cauthor_uid=17694058), [Kidd JR](http://www.ncbi.nlm.nih.gov/pubmed/?term=Kidd JR%5BAuthor%5D&cauthor=true&cauthor_uid=17694058), et al. Global diversity and evidence for coevolution of KIR and HLA. Nat Genet. 2007;39: 1114-1119.

**57**. Nemat-Gorgani N, [Edinur HA](http://www.ncbi.nlm.nih.gov/pubmed/?term=Edinur HA%5BAuthor%5D&cauthor=true&cauthor_uid=25139336), [Hollenbach JA](http://www.ncbi.nlm.nih.gov/pubmed/?term=Hollenbach JA%5BAuthor%5D&cauthor=true&cauthor_uid=25139336), [Traherne JA](http://www.ncbi.nlm.nih.gov/pubmed/?term=Traherne JA%5BAuthor%5D&cauthor=true&cauthor_uid=25139336), [Dunn PP](http://www.ncbi.nlm.nih.gov/pubmed/?term=Dunn PP%5BAuthor%5D&cauthor=true&cauthor_uid=25139336), [Chambers GK](http://www.ncbi.nlm.nih.gov/pubmed/?term=Chambers GK%5BAuthor%5D&cauthor=true&cauthor_uid=25139336), et al. KIR diversity in Maori and Polynesians: populations in which HLA-B is not a significant KIR ligand. Immunogenetics. 2014;66: 597-611.

**58**. Augusto DG, Piovezan BZ, Tsuneto LT, Callegari-Jacques SM, Petzl-Erler ML. KIR gene content in amerindians indicates influence of demographic factors. PLoS One 2013;8: e56755

**59**. Flores AC, Marcos CY, Paladino N, Capucchio M, Theiler G, Arruvito L, et al. KIR genes polymorphism in Argentinean Caucasoid and Amerindian populations. Tissue Antigens. 2007;69: 568-576.
